# Azithromycin plus chloroquine: combination therapy for protection against malaria and sexually transmitted infections in pregnancy

**DOI:** 10.1517/17425255.2011.598506

**Published:** 2011-07-07

**Authors:** R Matthew Chico, Daniel Chandramohan

**Affiliations:** London School of Hygiene and Tropical Medicine, Faculty of Infectious and Tropical Diseases, Disease Control Department, London, UK

**Keywords:** antental care, azithromycin, chloroquine, fetal, intermittent preventive treatment, malaria, maternal health, neonatal, pregnancy, reproductive tract infections, sexually transmitted infections, sub-Saharan Africa

## Abstract

**Introduction:**

The first-line therapy for the intermittent preventive treatment of malaria in pregnancy (IPTp) is sulphadoxine-pyrimethamine (SP). There is an urgent need to identify safe, well-tolerated and efficacious alternatives to SP due to widespread *Plasmodium falciparum* resistance. Combination therapy using azithromycin and chloroquine is one possibility that has demonstrated adequate parasitological response > 95% in clinical trials of non-pregnant adults in sub-Saharan Africa and where IPTp is a government policy in 33 countries.

**Areas covered:**

Key safety, tolerability and efficacy data are presented for azithromycin and chloroquine, alone and/or in combination, when used to prevent and/or treat *P. falciparum, P. vivax*, and several curable sexually transmitted and reproductive tract infections (STI/RTI). Pharmacokinetic evidence from pregnant women is also summarized for both compounds.

**Expert opinion:**

The azithromycin-chloroquine regimen that has demonstrated consistent efficacy in non-pregnant adults has been a 3-day course containing daily doses of 1 g of azithromycin and 600 mg base of chloroquine. The pharmacokinetic evidence of these compounds individually suggests that dose adjustments may not be necessary when used in combination for treatment efficacy against *P. falciparum, P. vivax*, as well as several curable STI/ RTI among pregnant women, although clinical confirmation will be necessary. Mass trachoma-treatment campaigns have shown that azithromycin selects for macrolide resistance in the pneumococcus, which reverses following the completion of therapy. Most importantly, no evidence to date suggests that azithromycin induces pneumococcal resistance to penicillin.

## Expert Opinion

1. Introduction
2. Leading candidates to replace SP in IPTp
3. Introduction to the compounds
4. Pharmacokinetics and pharmacodynamics
5. Regulatory affairs
6. Conclusion
7. Expert opinion

## 1. Introduction

Malaria infection in pregnancy is associated with low birth weight [1], preterm delivery [2], intrauterine growth-retardation [3] and maternal anemia [4]. An estimated 125 million pregnant women worldwide are at risk of malaria infection each year [5]. The World Health Organization (WHO) recommends the intermittent preventive treatment of malaria in pregnancy (IPTp) for pregnant women in areas of stable transmission [6] using a full-treatment course of 1.5 g sulphadoxine plus 75 mg pyrimethamine (SP) administered two to three times following the onset of fetal movement [6]. SP-IPTp is a government policy in 37 countries worldwide, 33 of which are in sub-Saharan Africa [7]. The recent decline in parasite sensitivity to SP makes identifying alternative therapies for use in IPTp an urgent priority [8]. Table 1 lists the characteristics of an optimal IPTp drug. While no candidates to replace SP match the ideal profile, azithromycin-chloroquine (Box 1) may be an attractive alternative for several reasons. Both azithromycin [9-12] and chloroquine [13-16] have been safely administered individually in all trimesters of pregnancy. The combination has demonstrated additive to synergistic effect *in vitro* [17] and *in vivo* [18] against *Plasmodium falciparum.* Clinical trials in sub-Saharan Africa have produced day-28 adequate parasitological responses (APRs) exceeding 95% (studies 82563, 367653 [81] and 82576/O26-44 [83]) after adjustment with polymerase chain reaction (PCR) methods, a threshold recommended by the WHO for new and/or alternative antimalarial therapy. A combination regimen with the higher azithromycin dose of 2 g is required to achieve > 95% parasitological clearance rates in India and South America. If used in IPTp, azithromycin may also provide protection against several sexually transmitted and reproductive tract infections (STI/RTI) including *Treponema pallidum* [19], *Neisseria gonorrhoeae* [20], *Chlamydia* trachomatis [21], *Trichomonas vaginalis* [22], and possibly bacterial vaginosis as observed with other broad-spectrum antibiotics administered in the first half of pregnancy [23]. This could be consequential as the combined prevalence of curable STI/ RTI is equal to, and higher in some settings, than the burden of malaria in pregnancy among women who seek antenatal care (ANC) in sub-Saharan Africa [24].

**Table 1:** Optimal IPTp drug profile.

The optimal IPTp therapy would be a combination of two molecules that:	
✓	Exhibit similar time above minimum inhibitory concentrations
✓	Support a once per month dosing regimen (≤ 2 doses)
✓	Have different mode of actions to reduce resistance selection
✓	Are active against asexual & sexual stages
✓	Are not necessarily rapid acting*
✓	Are either a fixed dose or loose combination
✓	Different from first line treatment for symptomatic malaria
✓	Ideally active against other treatable maternal health problems, e.g. STI/RTI, maternal-fetal transmission of infection
✓	Are safe during all the pregnancy, although pregnant women are unlikely to receive the first dose of IPTp in the first trimester
✓	Cost as little as possible per pregnancy with prices that are in line with current price estimates for artemisinin combination therapy (ACT)
*Note: There are no new classes of medicines with plasma exposures above MIC for 28 days. Such exposure is most likely to be achieved by slow release depot formulations which would require molecules with doses of less than 10 mg/day. No such compounds currently exist.*	

* Symptomatic women are treated with ACTs.

**Source:** Duparc, S. (2011). *Personal communication*. Medicines for Malaria Venture, Geneva, Switzerland.

**Box 1:** Drug summary

	
Drug name (generic)	Azithromycin-chloroquine
Phase	Phase III
Indication	Intermittent preventive treatment of malaria in pregnancy
Pharmacology description/mechanism of action	Azithromycin is a slow-acting macrolide that targets the 70S ribosomal subunit of the apical complex in susceptible microorganisms including malaria parasites Chloroquine is a rapidly absorbed 4-aminoquinoline that accumulates in digestive vacuoles, binds to hematin and prevents its expulsion from sensitive malaria parasites
Route of administration	Oral/Per os (PO)
Molecular formula	Azithromycin – C_38_H_72_N_2_O_12_
Chemical structure	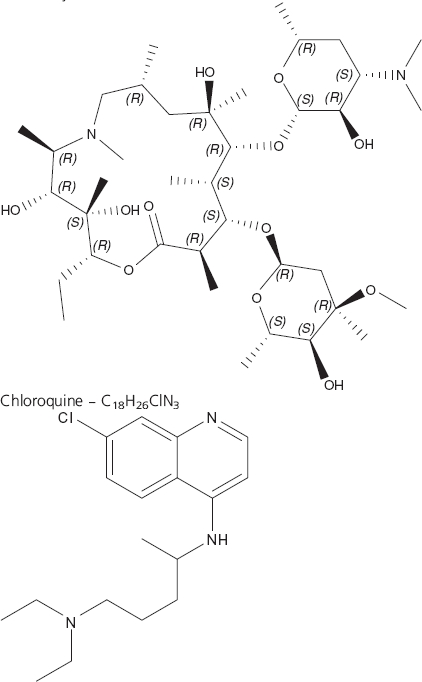
Pivotal trial(s)	ClinicalTrials.gov Identifier: NCT01103063 ‘Evaluate Azithromycin Plus Chloroquine And Sulfadoxine Plus Pyrimethamine Combinations For Intermittent Preventive Treatment Of Falciparum Malaria Infection In Pregnant Women In Africa’ [113]

This review consolidates evidence from peer-review publications, conference reports and abstracts, as well as data from a recently published Cochrane review of azithromycin for the treatment of uncomplicated malaria in non-pregnant adults and discusses the potential use of azithromycin plus chloroquine for IPTp.

## 2. Leading candidates to replace SP in IPTp

### 2.1 Mefloquine

Excluding selected areas of multidrug resistance along the Thai-Cambodian and Thai-Burmese borders, mefloquine is an efficacious chemoprophylaxis for the prevention of chloroquine-resistant *P.falciparum* [25] with a half-life between 14 and 41 days in healthy volunteers [26]. It is appealing as an IPTp drug, in part, because it can be administered as a single therapeutic dose, like SP, during ANC visits. Thus, policy change from SP to mefloquine could have minimal impact on ANC service delivery. However, the single-dose regimen has been associated with a high prevalence of adverse reactions among pregnant women in Malawi (750 mg) [27] and Benin (15 mg/kg) [28]. In Benin, adverse events were experienced by 78% of women following the first mefloquine-IPTp dose, of which 28% sought medical care for their side effects. A review of mefloquine treatment among 3673 patients of all ages living along the Thai-Burmese border found that the most important adverse effect was drug-induced vomiting within the first hour of ingestion [29]. Recently, a double-blinded, placebo-controlled trial of racemic mefloquine and (+)-mefloquine among healthy male and female volunteers in the UK was terminated prematurely due to high frequency of adverse events in both treatment groups. [30]. Severe central nervous reactions are of particular concern, occurring in ∼ 1 in every 6000 [31] to 10,000 [32] individuals. Women are affected by these reactions two times more often than men [33], a difference that may be attributable to dose-related toxicity that is more common among individuals with low body weight [34]. A Cochrane review of malaria chemoprophylaxis among travellers implicated mefloquine use at recommended dosages in the deaths of 22 travelers, including five suicides; no other drug had such reports [35]. Thus, despite its *P. falciparum* efficacy and ease of single-dose administration, it may be difficult to justify giving mefloquine to all women in IPTp regardless of their malaria status. Providing split-dose therapy may improve tolerability [36] but the long half-life of mefloquine raises the potential for drug-induced neuropsychiatric adverse events that persist for months [37].

### 2.2 Azithromycin-based combinations (other than azithromycin plus chloroquine)

Sulphadoxine-pyrimethamine plus azithromycin may be useful where there is a low-to-moderate prevalence of parasites with *dhfr/dhps* (dihydrofolate reductase/dihydropteroate synthase) mutations. However, evidence is inconclusive in locales with a high prevalence of quintuple-mutant parasites. Recrudescent malaria, for example, was less frequent in Malawi among pregnant women who received two courses of SP-IPTp plus azithromycin (1 g/day for 2 days; 4 g total) compared with SP-IPTp alone, but the prevalence of placental parasitemia was similar [38]. In Malawi, the APPLe study (Azithromycin for the Prevention of Preterm Labor) failed to observe a difference in the prevalence of preterm delivery and birth weight among pregnant women who received 1 g azithromycin two times during the antenatal period, along with SP-IPTp, when compared with SP-IPTp alone [39]. One reason may have been the study design. In both treatment groups, 7.1% of women were venereal disease research laboratory VDRL-positive and, consequently, were given 1 g benzyl penicillin; the WHO recommends 2.4 MU of benzathine penicillin G (BPG) for pregnant women [40]. It is unknown whether such a course of benzyl penicillin affects fetal syphilis and, despite possibly curing maternal infection, azithromycin has not been proven efficacious against congenital infection if fetal tissues have already been penetrated by *T. pallidum* [41]. Thus, inappropriate treatment for congenital syphilis in both groups could have limited the protective effect of azithromycin on preterm delivery.

In contrast to these findings, a trial in Malawi recently reported clear benefit from more frequent dosing with SP-IPTp and azithromycin. The incidence of preterm delivery was 17.9 versus 15.4% among women given two doses of SP-IPTp compared with women given monthly SP-IPTp (p = 0.32). With the addition of 1 g azithromycin on two occasions to monthly SP-IPTp, the incidence of preterm delivery was lowered further to 11.8% (p = 0.01). In this study, women who tested positive for syphilis were given 2.4 MU of BPG. Compared with the control group, women who received azithromycin also had a 35% lower risk of *T. vaginalis* infection (risk ratio = 0.65; p = 0.02) [22].

Piperaquine may possibly be combined with azithromycin and may be better tolerated than chloroquine [42], although the two have never been tested together and the teratogenicity of piperaquine is unknown. Further investigation is needed into the prolongation of the cardiac QTc interval observed following piperaquine treatment [43]. Pyronaridine and mefloquine have each demonstrated an additive effect *in vitro* with azithromycin [17], although pyronaridine teratogenicity requires evaluation. Dihydroartemisinin plus azithromycin are additive to synergistic *in vitro [44]* whereas artesunate has shown an antagonistic effect *in vitro* with azithromycin [17,45]. This is important as it may explain the poor *in vivo* efficacy observed in Tanzania during a pediatric trial that combined artesunate and azithromycin [46], but not seen in trials among semi-immune adults in Thailand [47] and Bangladesh [48]. The *in vitro* antagonism between azithromycin and artesunate may not have been apparent in Thailand and Bangladesh because the semi-immune adults were better able to render an acquired-immune response and, thus, overcame infection despite the drug antagonism, compared with more immunologically naive pediatric patients in Tanzania.

## 3. Introduction to the compounds

### 3.1 Azithromycin

#### 3.1.1 Safety and tolerability

Synthesized in the 1980s, azithromycin is the first compound of the azalide family of antibiotics. Animal studies have shown that quantities two to four times the human daily dose do not reduce fertility nor cause fetal harm [49]. Doses up to 2 g azithromycin have been used in all trimesters of human pregnancy. A one-time dose of 1 g azithromycin is associated with mild-to-moderate side effects in adults including diarrhea or loose stools (7%), nausea (5%), vomiting (2%), and vaginitis (2%) with < 1% experiencing dizziness, headache, vertigo and/or somnolence [49]. Azithromycin is better tolerated than erythromycin and can be taken for shorter time periods to achieve the same therapeutic effect [50]. Long-term azithromycin chemoprophylaxis among HIV-positive patients, however, may be poorly tolerated [51,52].

#### 3.1.2 Efficacy

The chemoprophylactic efficacy of azithromycin against *P. vivax* has been known for 15 years. A trial in Indonesia among civilians and soldiers with limited immunity showed that a loading dose of 750 mg azithromycin, followed by 250 mg/day, was 100% (95% CI: 83.9 - 100) and 98.3% (95% CI: 89.4 - 99.9) protective, respectively, against *P. vivax* during a 20-week period [53]. Comparable chemoprophylactic efficacy, 98% (95% CI: 88 - 100), was reported in a study among a similar population in Thailand [54]. Although these studies were conducted between 1996 and 1997, parasite sensitivity to azithromycin is likely to be the same today as the drug has not been used on any scale for malaria prevention.

Azithromycin is less active against *P. falciparum.* The trial in Indonesia described above reported 88.4% (95% CI: 56.6 - 97.4) and 62.9% (95% CI: 29.5 - 80.4) protective efficacy against *P. falciparum* in the same civilian and soldier populations, respectively, over a 20-week period [53]. A study of P. *falciparum* in India using a regimen of 1 g azithromycin plus placebo chloroquine on days 0, 1 and 2 produced an APR of 36% (5/14) at day 28 without PCR adjustment [18]. This was the first part of a two-stage trial that demonstrated *in vivo* synergy between azithromycin and chloroquine, and is described in greater detail within the azithromycin-chloroquine efficacy section.

### 3.2 Chloroquine

#### 3.2.1 Safety and tolerability

Chloroquine is a 4-aminoquinoline antimalarial drug. Antenatal dosing with hydroxychloroquine throughout pregnancy has shown to have no effect on newborns up to 1 year postpartum [55]. An observational study did not detect any ophthalmo-logical abnormalities in the children born to women who used hydroxychloroquine or chloroquine for a mean of 7.2 months during pregnancy [16]. The most commonly reported side effect of chloroquine in African population is pruritus which peaks 24 h after an oral dose [56]. Tolerability may vary among African populations as three times the treatment dose formerly recommended by the WHO does not appear to increase the incidence of adverse events in Guinea-Bissau [57]. Six times the therapeutic dose of 600 mg chloroquine can produce hypotension and cardiac failure [58]. Nevertheless, chloroquine is generally well tolerated in treatment doses, can be safely administered in any trimester of pregnancy [13-16] and readily crosses the placenta of pregnant women without teratogenic effect [59].

#### 3.2.2 Efficacy

Chloroquine was developed in 1934 and became the first-line treatment for all forms of malaria in the late 1940s and 1950s. A dose-finding study conducted during World War II reported that a regimen of 1500 mg base was used over 3 days to cure 10 Chinese and 8 American soldiers infected with *P. falciparum* along the India-Burma border [60], important in so much as scant evidence was used to set a treatment regimen that had only been slightly modified in WHO recommendations some 50 years later [61]. A recent systematic review of studies shows that chloroquine still has an APR of 92.3% (95% CI: 90.3 - 94.2) at day 28 against *P. vivax* [62]. Treatment failures, however, have been on the rise over the past 5 years [63] with the primary foci of resistance in Indonesia, Papua New Guinea, Timor-Leste and other parts of Oceania [64]. Reports of chloroquine resistance have also come from India [65] and South America [66,67].

Although chloroquine remains available at the community level in many settings, it is no longer recommended for the treatment of uncomplicated *P. falciparum* infection. Prior to the introduction of SP-IPTp, pregnant women commonly received sachets of chloroquine chemoprophylaxis during antental consultations, each containing four weekly doses of 300 mg for self-administration [68]. Even today, chloroquine may offer modest chemoprophylactic effect against low birth weight among pregnant women in West Africa [69], although this may be limited to multigravidae [70]. Table 2 contains efficacy data of four studies that included chloroquine monotherapy arms, two being pediatric treatment trials of particular note. These are described within their national contexts below.

**Table 2:** Efficacy of azithromycin, chloroquine or the combination against *P. Falciparum* infection observed among non-pregnant adults and children in recent studies.

Country	Years of study [Ref.]	Drug regimen	Sample size	PCR-unadjusted APR at day 28 (95% CI)	PCR-adjusted APR at day 28 (95% CI)
Western Kenya	2004 [81] Pfizer 82563	1 g AZ plus 600 mg CQ days 0, 1, 2	5	NA	100% (NA)
		600 mg CQ days 0, 1, 2 plus AZ placebo	7	NA	87.5% (59.8 – 100)
Malawi (children)	2005 [74] Laufer *et al*.	10 mg/kg CQ days 0, 1 and 5 mg/kg CQ day 2	80	NA	98.7% (96.3 – 100)*
		SP (1.25 mg/kg and 5 mg/kg) day 0	87	NA	18.4% (10.3 – 26.5)*
Ghana, Kenya, Mali, Uganda, Zambia	2004 – 2006 [83] O26-44 [81] Pfizer 82576	1 g AZ and 600 mg CQ days 0, 1, 2 plus MQ placebo day 0	103	NA	98.1% (95.4 – 100)
		500 mg AZ and 600 mg CQ days 0, 1, 2 plus MQ placebo day 0	Arm suspended: inadequate efficacy of regimen outside of Africa		
		750 mg MQ and 500 mg MQ day 0 plus placebo AZ and CQ days 0, 1, 2	103	NA	99.0% (97.1 – 100)
Burkina Faso, Ghana, Kenya, Mali, Senegal, Zambia	2006 – 2007 [81] Pfizer 367653	1 g AZ and 600 mg CQ days 0, 1, 2	107	NA	100% (NA)
		750 mg MQ and 500 mg MQ day 0	112	NA	99.1% (97.4 – 100)
Guinea-Bissau (children)	2006 – 2008 [79] Ursing *et al*.	50 mg/kg CQ in 6 doses days 0, 1, 2	158	NA	95.1%* (91.5 – 98.4)
		AL (20 mg/120 mg) up to 4 tablets at 0, 8, 24, 36, 48 and 60 h	168	NA	96.6%* (93.6 – 99.2)
India	1998 – 2001 [18] Dunne *et al*. 2005b	1 g AZ plus CQ placebo days 0, 1, 2	15	33.3% (9.5 – 57.2)*	NA
		600 mg CQ days 0, 1 plus placebo AZ day- 0, 1, 2 and CQ day 2	15	26.7% (4.3 – 49.1)*	NA
		1 g AZ days 0, 1, 2 and 600 mg CQ days 0, 1 and 300 mg CQ day 2	63	96.8% (92.5 – 100)*	NA
India	2004 – 2005 [129] O26-45 [81] Pfizer 74841	1 g AZ days 0, 1, 2 and 600 mg CQ day- 0, 1 and 300 mg CQ day 2	73	83.6% (75.1 – 92.1)	NA
		500 mg AZ and 600 mg CQ days 0, 1, 2 plus placebo 500 mg AZ days 0, 1, 2	59	66.1% (54.0 – 78.2)	NA
		600 mg CQ days 0, 1 and 300 mg CQ day 2 SP (1.5 g/75 mg) day 0	72	94.4% (89.2 – 99.7)	NA
Indonesia	2004 – 2005 [81] Pfizer 84240	1 g AZ and 600 mg CQ days 0, 1, 2 plus placebo SP day 0	13	NA	30.8% (5.7 – 55.9)‡
		500 mg AZ and 600 mg CQ days 0, 1, 2 plus placebo 500 mg AZ days 0, 1, 2 and placebo SP day 0	Arm suspended after 19 randomizations; no efficacy data reported		
		SP (1.5 g/75 mg) day 0 plus placebo 1 g AZ and placebo 600 mg CQ days 0, 1, 2	10	NA	80% (55.2 – 100)
Colombia and Suriname	2004 – 2005 [130] O26-46 [81] Pfizer 84227	1 g AZ days 0, 1, 2 and 600 mg CQ days 0, 1 and 300 mg CQ day 2	112	NA	60.1% (51.7 – 68.8)‡
		500 mg AZ and 600 mg CQ days 0, 1, 2 plus placebo 500 mg AZ days 0, 1, 2	Arm suspended: inadequate efficacy 36.4% (4/11) day-28 PCR-unadjusted		
		AP (1 g/400 mg) days 0, 1, 2	113		100% (NA)
Colombia and India	2006 – 2008 [84] ASTMH 62/374 [81] Pfizer 282919	2 g AZ and 600 mg CQ days 0, 1, 2	107	NA	97.2% (94.1 – 100)‡

* Appropriate clinical and parasitic response (ACPR).

‡ Partially PCR-adjusted.

AL: Artemether–lumefantrine (Coartem®); AP: Atovaquone–proguanil; AZ: Azithromycin; CQ: Chloroquine; MQ: Mefloquine; PCR: Polymerase chain reaction; PQ: Piperaquine; SP: Sulphadoxine–pyrimethamine.

##### 3.2.2.1 Malawi and parasite sensitivity to chloroquine

Malawi was the first country in sub-Saharan Africa to abandon chloroquine in favor of SP for the treatment of uncomplicated malaria. In 1993, chloroquine treatment failure rates had been as high as 57.8% [71]. Five years later, chloroquine *in vitro* testing inhibited blood schizont development in 96.5% (28/29) of isolates, suggesting that selection pressure for chloroquine-resis-tant polymorphisms in the *pfcrt* and *pfmdr 1* genes reduced as the use of chloroquine declined. Field sampling in 2001 failed to detect parasites with *pfcrt* [72] and chloroquine *in vivo* was 100% efficacious (63/63) in eradicating *P. falciparum* from asymptomatic semi-immune adults given 600 mg on days 0 and 1, and 300 mg on day 2 [73]. In 2005, a pediatric treatment regimen of 10 mg of chloroquine base/kg on days 0 and 1, and 5 mg/kg on day 2 produced a 98.88% (79/80) day 28 adequate clinical and parasitological response (ACPR) [74]. Genetic analysis of *P. falciparum* isolates between 1992 and 2005 suggests that chloroquine-susceptible parasites re-expanded their presence in Malawi after surviving undetected within asymptomatic hosts at the time drug pressure was removed [75]. Nevertheless, perpetuating chloroquine-resistant polymorphisms comes with a high fitness cost for *P. falciparum* [76], which remains the most likely explanation for the rapid return of sensitivity.

##### 3.2.2.2 Guinea-Bissau and the impotency of chloroquine resistance

*Plasmodium falciparum* resistance to chloroquine was first reported in Guinea-Bissau in 1990 [77]. Between 1992 and 2005, an estimated 33% of parasites (range 14 - 54%) obtained from asymptomatic children were chloroquine-resistant. Among these strains, *pfcrt* 76T was associated with resistance but *pfmdr 1* 86Y was not. In addition, the prevalence of single-nucleotide polymorphisms at *pfcrt* positions 76, 271 and 326, and *pfindrl* position 86 did not change significantly [78]. During the same time period in the same geographic areas, the median *3-day* chloroquine-treatment course was 63 mg/kg/day at health facilities, ranging from 60 mg/kg in 1995 to 75 mg/kg in 2000. Although the treatment efficacy was not reported, it appears as though a regimen with 2.5 times the WHO-recommended course suppressed the survival of *P. falciparum* and wild-type mutation [79]. This suggests that chloroquine resistance, while widespread, is not particularly potent [80].

Reinforcing this are data from a recent pediatric trial in Guinea-Bissau. A 3-day course of 50 mg/kg chloroquine, divided into six doses, was not inferior to a standard course of artemether-lumefantrine (20 mg/120 mg) or Coartem®, (Novartis) administered as up to four tablets at 0, 8, 24, 36, 48 and 60 h. The day-28 PCR-adjusted ACPR was 95.1% (150/158) for chloroquine and 96.6% (162/168) for artemether-lumefantrine. The PCR-adjusted ACPR for chloroquine was 93.8% (138/148) at day 42 and 93.1% (114/125) at day 70 [82]. The PCR-adjusted ACPR among the 60 patients with *P. falciparum* strains containing *pfcrt* 76T at days 28, 42 and 40 were 86.7, 82.3 and 79.7%, respectively [79]. No severe drug-related adverse events were reported, although pruritus was reported in 19.9% (36/181) of children in the chloroquine group compared with 5.4% among those given artemether-lumefantrine (p < 0.001).

### 3.3 Azithromycin plus chloroquine

#### 3.3.1 Safety and tolerability

Data available on the safety and tolerability of azithromycin plus chloroquien are limited. However, the analysis in the Cochrane review shows a dose-response relationship with azithromycin and nausea; 33% (33/100) of participants given a 3-day course containing 2 g/day azithromycin reported nausea, compared with 9.6% (11/114) from a 3-day regimen of 1 g/day azithromycin. No other dose-response relationships were observed, although chloroquine-associated pruritus was common in sub-Saharan Africa studies where the prevalence ranged from 28.3% (32/113) to 51.8% (59/114) [81]. Data are not disaggregated by country or study site, but as previously noted, evidence from a study among children in Guinea-Bissau suggests that some African populations tolerate chloroquine better than others [79].

#### 3.3.2 Efficacy

A placebo-controlled two-arm trial followed by an open-label single-arm study in India demonstrated *in vivo* synergy using the combination of azithromycin plus chloroquine [18]. In the placebo-controlled trial, 32 semi-immune subjects were treated for uncomplicated *P. falciparum* malaria with either: (a) 1 g azithromycin plus chloroquine placebo for 3 days, or (b) 600 mg chloroquine the first 2 days and 300 mg on the last day, plus azithromycin placebo all 3 days. In the second open-label study, *64* semi-immune subjects with *P. falciparum* infection were treated with azithromycin and chloroquine using doses similar to the two-arm trial. ACPR in azithromycin without PCR correction at day 7 in the azithromycin monotherapy arm was 62.5% (10/16) whereas in the chloroquine monotherapy group it was 87.5% (14/16). By day 28, azithromycin had continued to suppress fever and parasites in only 33.3% (5/15) of subjects whereas chloroquine maintained an ACPR in just 26.7% of cases (4/15). These outcomes were in contrast to the treatment effect reported among subjects who received combination therapy. ACPR at day 7 was 96.8% (61/63) and the same level of ACPR was maintained at day 28. The observed difference in ACPR between the two studies may have been exaggerated because baseline parasite counts were three times higher among subjects in the randomized trial compared with the single-arm open-label study; mean parasite densities were 17,254 parasites/μl among those given azithromycin, 18,542 parasites/μl for chloroquine recipients, whereas the azithromycin plus chloroquine group had a mean of 6417 parasites/μl. However, this baseline difference may not be consequential in the context of IPTp; baseline mean parasite counts in several recent studies of pregnant women across treatment groups were 1154 per μl in Benin [28], 945 per μl in Malawi [38] and 194 per μl in Ghana [82]. It is also likely that women in sub-Saharan have greater acquired immunity than their Indian counterparts. Thus, the synergy between azithromycin and chloroquine observed in the Indian studies may be replicable elsewhere among pregnant women whose parasite densities at the time of treatment are equal to or lower than levels reported in the Indian study population.

The results of several published clinical trials, conference presentations and data from a recently published Cochrane review are consolidated in Table 2, while important contextual factors are discussed below. Studies investigating azithromycin plus chloroquine involved only non-pregnant adults with *P. falciparum* infections.

The first study of azithromycin plus chloroquine in sub-Saharan Africa was a small two-arm placebo-controlled trial in a high-transmission area of western Kenya that was suspended prematurely due to logistical issues. Nevertheless, a regimen of 1 g azithromycin plus 600 mg chloroquine daily for 3 days was able to achieve parasite eradiation in five of five subjects by day 28; one treatment failure (RIII) was reported that cleared by day 7 and did not recur through day 28 [81]. This was followed up by a multicenter, multicountry, placebo-controlled trial in sub-Saharan Africa that compared two regimens of azithromycin (1 g vs. 500 mg) plus 600 mg chloroquine daily on days 0, 1 and 2 against a split dose of mefloquine, 750 mg and then 500 mg 6 - 10 h later, administered on day 0. The study arm of 500 mg azithromycin was suspended early based on the data from South America and India; no data were reported. However the day-28 PCR-adjusted APR for the group given three doses of 1 g azithromycin plus 600 mg chloroquine was 98.1% (101/103) compared to mefloquine with 99.0% (102/103) [81]. Of particular interest is the sub-analysis of APR by *pfcrt* prevalence by the study site. In Ndola, Zambia, where the prevalence of *pfcrt* was 27%, the APR at day 28 was 100% (55/55); in jinja and Kampala, Uganda, where *pfcrt was* 98%, the APR at day 28 was 94.4% (17/18) [83]. A confirmatory multicenter trial comparing the same regimens of azithromycin-chloroquine and mefloquine *sans* placebo showed that the day-28 PCR-adjusted APR for azithromycin-chloroquine was 100% (107/107) and 99.1% (111/112) for mefloquine [81].

Several studies of azithromycin-chloroquine have been conducted outside of sub-Saharan Africa and are included in Table 2. Based on trials conducted in India, Indonesia and Colombia/Surinam, regimens containing 500 mg azithromycin plus 600 mg chloroquine for 3 days may contain insufficient azithromycin to achieve 95% APR at day 28. Similarly, regimens of 3 days containing < 600 mg chloroquine each day (300 mg on day 2, for example) may also not be able to reach the WHO-recommended treatment efficacy threshold. A multicenter study in India and Colombia evaluated a combination of 2 g azithromycin with 600 mg chloroquine base once daily for 3 days; the PCR-adjusted day-28 efficacy was 97.2% (104/107) [81,84].

## 4. Pharmacokinetics and pharmacodynamics

### 4.1 Azithromycin

Azithromycin is an analog of erythromycin, modified by the insertion of a nitrogen atom into the macrolide nucleus. It is stable at gastric pH with a high affinity for tissue due to the presence of two basic tertiary amine groups which enhance its amphiphilic properties [85]. Azithromycin targets the 70-S ribosomal subunit of the apical complex in susceptible microorganisms including *P. falciparum* and *P. vivax* [86]. Once attached, azithromycin hinders polypeptide development by triggering premature detachment and movement along the peptide exit tunnel. Thus, azithromycin induces ‘delayed death’ by either inhibiting genetic translation and causing the progeny of parasites to inherit non-functioning apicoplast [86-89] or rendering second-generation parasites incapable of establishing parasitophorous vacuoles following erythrocytic invasion [86].

Azithromycin accumulates in hepatic, renal, pulmonary and splenic tissue [90], slowly reaching the circulatory system over a 1-week period [86]. It has a half-life of 68 h in healthy volunteers [91] and an absolute bioavailability between 34 and 52% following oral administration [92,93]. Less than 3.0% of a maternal dose perfuses the placenta [94]. Azithromycin is not known to cause any clinically significant interactions [95].

**Figure 1:**
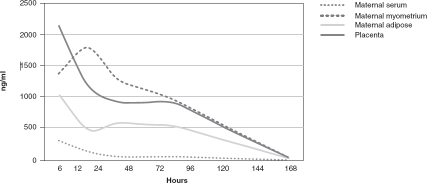
Pharmacokinetic elimination of 1 g azithromycin through maternal serum and myometriai, adipose and placental tissue (ng/ml/h). Adapted from [96].

A study of 20 pregnant women showed that maternal serum concentrations peak within 6 h of dosing and high serum concentrations are sustained for 24 h [96]. Compared with serum, azithromycin achieves high and sustained concentrations in the body tissues. In the above study, the concentrations were seven, six and three times higher in placental, myometrial and adipose tissues, respectively. Figure 1 illustrates the concentration-time profile of azithromycin over time in serum and tissues. A more recent pharmacokinetic study of azithromycin (two 2 g doses 24 h apart) plus chloroquine (450 mg base daily for 3 days) given to 31 pregnant and 29 non-pregnant women in Papua New Guinea showed that plasma concentrations of azithromycin differ between groups within the first 48 h of dosing. The pharmacokinetic profiles were similar between groups, indicating that dose adjustments may not be necessary among pregnant women, even in the presence of parasitemia [97]. Chloroquine pharmacokinetic end points were not reported.

### 4.2 Chloroquine

Chloroquine is quickly absorbed and reaches high concentrations in the digestive vacuoles of malaria parasites. Once there, chloroquine forms a complex with ferriproporphyrin IX (FP), a major toxic by-product of parasitic hemoglobin digestion, preventing parasites from polymerizing FP into harmless hemozoin and expelling it through their digestive vacuoles. As a result, parasite membranes become highly permeable, causing rapid death [98]. As discussed previously, resistance to chloroquine is associated with parasite protein *pfcrt* (mutant alleles K76T or, in two single cases, K76N or K76I [99]). These are located in the digestive membrane of the food vacuole [100,101]. Some researchers suspect that *pfcrt* enables protonated chloroquine to escape the food vacuole whereas others postulate that *pfcrt* binds directly to chloroquine, inhibiting its ability to alter vacuole pH [102].

Peak plasma concentrations of chloroquine are reached within 2 h of oral dosing with an absolute bioavailability ranging from 70 to ∼ 100% [103-105]. Chloroquine accumulates extensively in hepatic, connective and pigmented tissues [106]. Greatest concentrations are found in erythrocytes, granulocytes and platelets, whereas 55% is protein-bound in plasma [106]. Its half-life is 1 - 2 months [107,108].

Known pharmacokinetic interactions of chloroquine are presented in Table 3. Of note, chloroquine reduces the systemic exposure of praziquantel by 65% and peak concentrations by 59% [109]. Praziquantel is the first-line anti-schistosomal therapy recommended by the WHO for use among pregnant women in endemic areas [110].

**Table 3:** Drug interactions with chloroquine.

Drug	Type	Interaction with chloroquine	Clinical significance
Chlorpromazine	Antipsychotics	Chloroquine, SP and amodiaquine increase serum concentrations of chlorpromazine 1.7 – 4.3 times [131]	Unknown
Cimetidine	Histamine h2-receptor antagonist	Cimetidine increases serum concentration of chloroquine prolonging its half-life 48% [132]	Unknown
Codeine	Opiate	Chloroquine inhibits CYP2D6 and may theoretically interfere with bio-activation of codeine to morphine [133]	Unknown
Cyclosporine	Immunosuppressant	Cyclosporine concentrations increase up to 4.3 times when used with chloroquine [134]	Cyclosporine dosages may need to be reduced during concomitant chloroquine use
Kaolin-Pectin	Antidiarrhoeal	Kaolin–Pectin reduces the area under the plasma-chloroquine concentration–time curve by ∼ 30% [135]	Unknown
Methotrexate	Antimetabolite and antifolate	Methotrexate peak concentrations are reduced 20% and its area under the concentration–time curve is reduced 28% with concomitant chloroquine use [136]	Unknown
Praziquantel	Anthelmintic	Concomitant use of chloroquine reduces concentration–time curve of praziquantel 65% and peak concentrations 59% [109]	WHO recommends inclusion of pregnant women in deworming campaigns with 40 mg/kg praziquantel [110]

A pharmacokinetic study in Thailand among 12 pregnant and 13 non-pregnant women who received a 3-day course of 25 mg/kg chloroquine for acute *P. vivax* malaria reported that the total area under the whole-blood chloroquine concentration-time curve tended to decrease with gestational age. However, pregnancy did not alter overall pharmacokinetics and researchers concluded that no adjustment in regimen would be required for pregnancy [111]. The results of another pharmacokinetic study in Papua New Guinea involving 30 pregnant and 30 non-pregnant indicate otherwise. Women were given a daily dose of 450 mg chloroquine base for 3 days along with SP-IPTp per national policy. Chloroquine and related metabolites were still present 42 days later, but plasma concentrations were significantly lower in pregnant women. This may explain treatment outcomes among those with asymptomatic parasitemia at enrollment. In total, 43.3% (26/60) had malaria infections: 20 *P. falciparum,* 4 *P. vivax,* and 2 *P. malariae.* By day 28, *P. vivax* and *P. malariae* cases were cured whereas recrudescent *P. falciparum* was found among 5 of 13 pregnant women and 2 of 7 non-pregnant women. Thus researchers suggested a dose of 600 mg/ day among pregnant women, particularly important where *P. falciparum* is prevalent. Table 4 combines key pharmacokinetic results from the two Papua New Guinea studies, comparing selected end points for chloroquine [112] and azithromycin [97]. A Phase III multicenter study in sub-Saharan Africa is testing a fixed-dose combination of azithromycin-chloroquine for use in IPTp. The regimen contains 27% more chloroquine (620 mg base daily for 3 days) per course than the amount used in Papua New Guinea with the addition of 1 g azithromycin daily for 3 days [113]. A separate clinical trial being conducted in parallel at the same sites will evaluate the parasite clearance rates and pharmacokinetics of the same fixed-dose combination regimen in pregnant women with *P. falciparum* parasitemia [114].

**Table 4:** Chloroquine pharmacokinetics and azithromycin pharmacokinetics among pregnant and nonpregnant women in Papua New Guinea.

	Chloroquine pharmacokinetic study* [112]			Azithromycin pharmacokinetic study‡ [97]		
		
	Pregnant women (n = 30) [95% CI]	Non-pregnant women (n = 30) [95% CI]	p	Pregnant women (n = 31) [95% CI]	Non-pregnant women (n = 29) [95% CI]	p
*Parameter*						
CL/*F* (l/h)	32.0 [28.8 – 36.5]	23.9 [21.3 – 26.3]	< 0.001			
CL_M_/*F* (l/h)	5.74 [5.17 – 6.55]	4.29 [3.82 – 4.72]	< 0.001			
*V_c_*/*F* (l)	3406 [2819 – 4919]	2702 [2230 – 3535]	0.007	647 [422 – 995]	249 [157 – 363]	NS
*V_P2_*/*F* (l)				3888 [3708 – 4104]	3672 [3456 – 3888]	0.034
*V_ss_*/*F* (l)	7147 [6721 – 9638]	6707 [5843 – 7158]	0.009	8355 [7460 – 8973]	6875 [6115 – 7526]	0.002
*t*_1/2α_ (h)				0.88 [0.57 – 1.36]	0.39 [0.24 – 0.56]	< 0.001
*t*_1/2β_ (h)	266 [244 – 280]	291 [272 – 313]	< 0.001	20.7 [18.3 – 22.8]	18.8 [15.3 – 21]	NS
*t*_1/2γ_ (h)				78.2 [74 – 82.5]	77.1 [71.5 – 84.5]	NS
AUC_0 – ∞_	35,750 (mg · h/litre) [31,343 – 39,729]	47,892 (mg · h/litre) [43,486 – 53,746]	< 0.001	28,713 (μg h litre^-1^) [25,913 – 32,942]	31,781 (μg h litre^-1^) [28,736 – 38,012]	NS

* Chloroquine pharmacokinetic study: all women received 450 mg base chloroquine for 3 days plus SP-IPTp.

‡ Azithromycin pharmacokinetic study: women received either two 2 g doses azithromycin plus 450 mg base chloroquine for 3 days OR two 2 g doses azithromycin plus SP-IPTp.

AUC_0 – ∞_: Area under the curve; CL/*F*: Clearance from the first compartment/bioavailability; CL_M_/*F*: Metabolic clearance/bioavailability; NS: Not significant;

*t*_1/2α_: First distribution half-life; *t*_1/2β_: Elimination half-life; *t*_1/2γ_: Terminal half-life; *V_c/F_*: Volume of distribution of the first compartment/bioavailability; *V_P2/F_*: Volume of distribution of the second compartment/bioavailability; *V_ss_/F*: Volume of distribution at steady state/bioavailability.

### 4.3 Azithromycin plus chloroquine

Azithromycin and chloroquine do not exhibit any direct pharmacokinetic interactions [115]. Chloroquine is known to delay cardiac repolarization through inhibition of the potassium ion channel [116], increasing the chances of prolonging the electrocardiogram QT interval, while azithromycin does not [117]. The assessment of electrical alternans in an anesthetized guinea-pig showed that there is no additional risk of arrhythmia when azithromycin and chloroquine are used in combination; azithromycin may even be slightly protective of arrythmogenic risk when administered with chloroquine [118].

Re-emerging chloroquine sensitivity has been reported where its use has been suspended [73,119]. Given that azithromycin and chloroquine target unique metabolic pathways in *Plasmodia,* it is possible that the re-introduction of chloroquine with azithromycin as a partner drug may prevent re-selection of parasites carrying the *pfcrt* mutation. This must be verified in appropriate clinical trials and monitored with ongoing surveillance. Azithromycin use against trachoma in mass-treatment campaigns has induced transient resistance in the pneumococci [120]. A cluster-randomized trial for trachoma control in Ethiopia reported that four treatments of 1 g azithromycin over a 1-year period among children aged 1-5 years of age increased the prevalence of azithromycin resistance in pneumococcal isolates from 6.3% (95% CI: 1.0 - 15.7) to 62.3% (95% CI: 49.1 - 75.4), a full year after the final course of azithromycin has been administered [121]. Communities were not followed after treatments were stopped, but single-dose mass administration campaigns have reported rapid increases in azithromycin resistance among pneumococcal isolates, only to have them return to baseline levels within 6-12 months [120,122]. Data from Ethiopia shows similar effects on the pneumococci following mass dosing of children with 1 g azithromycin every 6 months. After 3 years and six courses of azithromycin, 76.8% (95% CI: 66.3 - 85.1) of children carried azithromycin-resistant pneumococci, levels dropped rapidly following the cessation of dosing. Most importantly, however, is that no evidence appears to associate azithromycin use with the selection of penicillin resistance among the pneumococcus. It is unknown whether macrolide resistance will persist in the context of IPTp. Monitoring of resistance markers will be important if AZCQis adopted for antenatal use. The dosing regimen of AZCQ, containing 3 g azithromycin (1 g daily for 3 days), may possibly be counterselective and curb wild-type pneumococci survival; evidence from a 10-year multinational surveillance study shows that the treatment of respiratory tract infections to the point of bacterial eradication minimizes the potential for selecting and maintaining resistant strains [123].

The potential for developing azithromycin resistance in syphilis is possible as has been observed in high-income countries [124]. This is considerably less likely to occur in sub-Saharan Africa if pregnant women with syphilis are simultaneously given BPG along with azithromycin-chloroquine.

## 5. Regulatory affairs

The combination therapy azithromycin-chloroquine is not currently registered for any indication, although Pfizer may pursue an application for IPTp. SP and mefloquine are both manufactured by Roche Pharmaceuticals which is not known to be preparing a registration dossier for the use of either antimalarial therapy in pregnancy.

## 6. Conclusion

Azithromycin-chloroquine is a potential alternative to SP, having shown efficacy against *P. falciparum* among non-pregnant adults in sub-Saharan Africa, Colombia and India, even in the presence of parasite populations saturated with chloroquine-resistance markers. The combination may be safely administered any time in pregnancy and offers benefits of clearing several STI/RTI. Pharmacokinetic measurements in pregnancy suggest that dose adjustments may not be necessary for azithromycin but daily chloroquine dosing needs to be 600 mg for 3 days.

## 7. Expert opinion

Malaria transmission has declined in some epidemiological settings. There is no evidence to suggest, however, that the risk of malaria in pregnancy without preventive measures has declined in the same locations. It is possible that the risk of adverse events associated with malaria in pregnancy will increase for an unknown period of time while malaria control and elimination measures are scaled up and multigravidae fail to acquire immunity through exposure in earlier pregnancies. Thus, the need to identify a replacement for SP is as important as ever.

Therapy that combines antimalarial and antimicrobial protection, and safe administration in any trimester of pregnancy, is essential to the profiles of drugs that may replace SP for IPTp. This is important for three reasons: i) the prevalence of malaria and curable STI/RTI in pregnancy is similar and the burden of STI/RTI will increase, proportionately, as malaria control measures and elimination measures are scaled up; ii) peripheral parasitemia is actually highest between gestational weeks 9 and 16, a period when SP and all other compounds currently under investigation, except azithromycin-chloroquine, would be largely contraindicated [1,125]; and iii) *T. vaginalis* and the bacteria associated with bacterial vaginosis are believed to trigger preterm delivery by slowly secreting proteases that degrade and weaken fetal membranes [126,127]. Subanalysis in a Cochrane review illustrates the importance of early intervention: in five trials of 2387 women who were treated before 20 weeks gestation, the use of antibiotics was associated with a statistically significant decreased risk of preterm birth < 37 weeks (Peto Odds Ratio 0.72, 95% CI: 0.55 - 0.95) [23]. These encouraging observations need to be verified by further clinical study as the pooled data did not include head to head comparisons of early versus late treatment. Nevertheless, because azithromycin-chloroquine is safe in all trimesters, healthcare providers may be less concerned about imprecise gestational estimates in pregnancy while administering this combination of drugs as IPTp. Treatment compliance, however, will rely on pregnant women self-administering drugs. Co-formulated tablets being tested for IPTp are film-coated and, thus, may not have a bitter taste; sugar-coating chloroquine tablets increased the use of monotherapy by *64%* [128] and could be considered for azithromycin-chloroquine.

There is some concern that the use of azithromycin-chloroquine in IPTp may increase the prevalence of azithromycin-and erythromycin-resistant pneumococci, although evidence from mass trachoma-treatment campaigns suggests that the selection for resistant mutations is transient in non-pregnant participants. This needs to be monitored as part of any IPTp program.
